# Sleep-Disordered Breathing in Children with Cerebral Palsy Compared to Non-Neurological Controls: A Prospective Study from a Tertiary Center in Jordan

**DOI:** 10.3390/neurolint18030049

**Published:** 2026-03-02

**Authors:** Montaha Al-Iede, Abdallah Al-Ani, Amal Abu Libdeh, Amira Masri, Ahmad T. Qatawneh, Basim Alqutawneh, Nihad A. Almasri

**Affiliations:** 1 Division of Respiratory and Sleep Medicine, Department of Pediatrics, Jordan University Hospital, Amman 11942, Jordan; 2 School of Medicine, The University of Jordan, Amman 11942, Jordan; a.abulibdeh@ju.edu.jo (A.A.L.); amasri@ju.edu.jo (A.M.); ahmad.t.qatawneh@hotmail.com (A.T.Q.); 3 Division of Vascular and Interventional Radiology, Boston Children’s Hospital, Boston, MA 02115, USA; abdullah.al-ani@childrens.harvard.edu; 4 Division of Neurology, Department of Pediatrics, Jordan University Hospital, Amman 11942, Jordan; 5 Department of Radiology, Blacktown & Mount-Druitt Hospitals, Blacktown P.O. Box 792, Australia; bqutawneh@yahoo.com; 6 Department of Rehabilitation Sciences, College of Health Sciences, Qatar University, Doha P.O. Box 2713, Qatar; nalmasri@qu.edu.qa

**Keywords:** cerebral palsy, Jordan, polysomnography, pediatric sleep questionnaire, sleep disturbance scale for children, obstructive sleep apnea

## Abstract

Background/Objectives: Our aim was to evaluate sleep quality and the prevalence of OSA among children with CP and other neurological conditions using both polysomnography (PSG) and validated sleep questionnaires. Methods: This study was conducted at the sleep laboratory of the Jordan University Hospital (JUH) between October 2023 and April 2025. Patients were consecutively recruited from pediatric neurology clinics. Patients completed a PSG session, while guardians/caregivers completed the pediatric sleep questionnaire (PSQ) and sleep disturbance scale for children (SDSC) tools on the behalf of the included patients. The cohort was matched with a control group composed of asthmatic children referred for sleep disturbances. Results: We recruited 296 patients, of whom 41.6% had neurological disorders and 58.4% were matched non-neurological controls. Among those with neurological diseases (n = 123), 46.3% were diagnosed with CP. Patients with CP showed significantly lower sleep efficacy than controls (*p* < 0.001). They also had reduced total sleep time compared to non-neurological controls but, notably, longer sleep time than children with Down Syndrome (all *p* < 0.05). Patients with CP had arousal index and apnea–hypopnea index values comparable to controls, but both measures were significantly lower than those observed in children with Down Syndrome and other syndromic patients (all *p* < 0.05). The risk of OSA according to the PSQ was insignificant for both controls and patients with neurological conditions (OR: 0.898; *p* = 0.720). According to the SDSC questionnaire, patients with neurological conditions had a significantly higher risk of sleep disturbances compared to controls (OR: 2.015; *p* = 0.043). Conclusion: Patients with neurological diseases are at a higher risk of sleep disturbances compared to controls. This was significantly more apparent in non-CP patients than in those with CP. At present, PSG remains the most objective and reliable tool to evaluate these disturbances.

## 1. Introduction

Cerebral palsy (CP) is a heterogeneous group of motor impairment syndromes caused by non-progressive lesions in the developing brain [1]. According to Rosenbaum et al., motor disorders in CP are often accompanied by disturbances in sensation, perception, cognition, communication, and behavior, as well as epilepsy and secondary musculoskeletal problems [2]. Over 18 million people globally are affected by neurological dysfunction secondary to CP, with most cases primarily occurring during fetal development [3,4,5]. Despite the condition resulting from a non-progressive disturbance in the developing brain, individuals with CP experience ever-increasing challenges and quality of life disturbances due to growth, aging, or secondary complications of the disease [2].

Individuals with CP experience a wide range of complicated and overlapping symptoms [4,6,7]. These include pain, intellectual incapacity, speech and motor difficulties, epilepsy, incontinence, blindness, behavior limitations, and sleep disturbances. The latter is considered an important comorbidity, as it is associated with the type of CP and severity of neurological deficit [8]. According to Novak et al., one in five children with CP has a sleep disorder [9]. This link is postulated to be caused by CP-associated pain, mobility impairment, respiratory problems, drooling and uncoordinated swallowing, and sensory processing challenges [10,11]. The literature shows that 13 to 85% of children with neurodevelopmental disorders experience a clinically significant sleep disturbance [10]. These disturbances are a burden on both the affected individuals and their caregivers in terms of cognitive and psychological well-being [3,12,13,14].

Early identification and management of sleep disorders in children with neurological diseases, specifically CP, is crucial for sustaining quality of life, decreasing neurobehavioral consequences, improving symptom control, and preventing extended morbidity associated with sleep disorders (e.g., obstructive sleep apnea (OSA)) [8]. Few studies have examined how sleep disturbances vary across different neurological conditions or compared objective sleep measures in these populations. Therefore, the purpose of this study was to address this gap by evaluating sleep quality and the prevalence of OSA among children with CP, other neurological disorders, and typically developing controls, using both polysomnography (PSG) and validated sleep questionnaires.

## 2. Materials and Methods

### 2.1. Patient Recruitment and Data Collection

This study was conducted at the sleep laboratory of the Jordan University Hospital (JUH) between October 2023 and April 2025. JUH is a 600-bed tertiary teaching hospital with a two-bed sleep laboratory. The sleep laboratory performs both adult and pediatric sleep studies at a rate of 5 nights per week for adult sleep studies and 1 night per week for pediatric sleep studies.

Patients were consecutively recruited from the Pediatric Neurology Clinics at JUH, which are staffed by one of two full-time pediatric neurologists. A research assistant, who was funded by the university’s scientific research department, was responsible for recruiting eligible patients during their routine clinic visits. Parents or caregivers were asked to complete the pediatric sleep questionnaire (PSQ). Children who screened positive for sleep problems based on PSQ results and their clinical history were referred to the sleep laboratory for an overnight PSG study. Electronic medical records were searched retrospectively, and the following data was extracted: neurological diagnosis, type, and GMFCS grade for patients with CP, family history, anthropometric measures, and previous medications. All included patients underwent PSG and completed the PSQ and sleep disturbance scale for children (SDSC) questionnaires. Included participants were grouped into those with CP and those with other neurological conditions. The cohort of patients with neurological disorders was compared against a control group composed of patients attending the pediatric respiratory clinics.

### 2.2. Control Group Recruitment

The control group was formed of children who underwent overnight PSG in the pediatric sleep laboratory during the same period that patients with neurological conditions were being recruited. These children had no history of neurological disease and were matched to the study group by age and sex. Those children had well-controlled asthma and had been referred for a PSG study because of symptoms suggestive of OSA.

### 2.3. Polysomnography

PSG was conducted by certified technicians utilizing the Phillips Alex 6 equipment (Phillips, Amsterdam, The Netherlands). The results of the PSQ were examined by sleep medicine specialists at the sleep laboratory of JUH. Included participants underwent PSG within 1 h of their usual bedtime. The study used bilateral electrooculogram channels, namely the LOC–A1 and ROC–A1 channels, as well as submental electromyogram channels. The respiratory channels consisted of a nasal cannula, a pressure sensor for airflow monitoring, and plethysmography for monitoring breathing movements of the chest and belly. The levels of oxygen saturation (SaO2) and pulse were measured using a pulse oximeter. The PSG values were complemented by key blood gas parameters, including pH, partial pressure of carbon dioxide, bicarbonate concentration, and amount of base excess. The detailed extracted sleep characteristics were delineated in a previous publication [15].

### 2.4. Questionnaires Used

The guardians of included patients completed the PSQ and SDSC questionnaires. We utilized the Arabic version of the PSQ [16]. The questionnaire includes 22 items which are answered on a 3-point scale (affirmative, negative, or do not know). The total score was calculated by dividing the number of positive answers by the total count of items receiving either positive or negative responses, which ranged from 0 to 1. The denominator excludes items with missing replies and those answered with “do not know.” Scores exceeding 0.33 suggest a heightened risk of a sleep-related respiratory issue in children [17]. Similarly, we utilized the Arabic version of the SDSC scale [18]. The tool consists of 26 items answered on a 5-point Likert scale. Scores are generated by generating the sum of responses across all items, whereby higher scores indicate more acute sleep disturbances. A cut-off of 39 is considered to be the most sensitive and specific for screening for sleep disturbances [19].

### 2.5. Ethical Approval

This study adheres to ethical guidelines and ethical approval for this project was provided by the Institutional Review Board (IRB) of University of Jordan (IRB #:10/2023/2653). This study was done in accordance with the Declaration of Helsinki. Before starting the survey, we explained the aim of the study and the survey for the parents. Informed consent was obtained from the parents or legal guardians.

### 2.6. Statistical Analysis

All analyses were conducted using SPSS version 23.0.0. Categorical variables were presented as frequencies and associated percentages. Percentages were only calculated for participants with available data. Continuous variables were presented as either means ± standard deviations or medians with [interquartile range]. Associations between categorical variables were examined using the chi-squared test. Mean differences in continuous variables were explored using the Mann–Whitney U or Kruskal–Wallis tests, where applicable. Figures were produced using GraphPrism software version 8.4.3. A *p*-value of less than 0.05 was considered statistically significant.

## 3. Results

### 3.1. Characteristics of the Included Sample

A total of 296 pediatric patients were included in the final analysis. Patients with neurological disorders comprised 41.6% of the sample compared to controls with no neurological disease (58.4%). The two groups were matched for age (*p* = 0.252) and gender (*p* = 0.198). Among those with neurological diseases (n = 123), 46.3% were diagnosed with CP. Among CP patients (n = 57), 86.0% were quadriplegic, 8.8% were hemiplegic, while 5.3% had ataxia. For this cohort, their movement ranks were as follows: level 5 in 82.5% of patients, level 4 in 3.5%, level 2 in 8.8%, and level 1 in 5.3%. Table 1 showcases the participants’ characteristics.

### 3.2. Polysomnography Findings

Table 2 demonstrates the PSG values stratified by neurological disease status. Compared to controls, patients with neurological conditions demonstrated significantly lower sleep efficacy (*p* < 0.001), total sleep time (*p* < 0.001), awake arterial O_2_ saturation (*p* = 0.003), and Nadir of O_2_ saturation (*p* = 0.037). On the other hand, they demonstrated significantly higher N1 values (*p* = 0.005), a higher arousal index (*p* = 0.005), a greater apnea–hypopnea index (AHI) (*p* = 0.007), and greater levels of hypopnea (*p* = 0.001).

Table 3 presents PSG differences stratified by neurological conditions and compared to non-neurological controls. Patients with CP showed significantly lower sleep efficacy than non-neurological controls (*p* < 0.001). They also had reduced total sleep time compared to non-neurological controls but, notably, a longer sleep time than children with Down Syndrome (all *p* < 0.05). Moreover, patients with CP exhibited significantly lower N1 values compared to patients with neuromuscular conditions (*p* = 0.006).

Patients with CP had arousal index and AHI values comparable to those of non-neurological controls, but both measures were significantly lower than those observed in children with Down Syndrome and other syndromic patients (all *p* < 0.05). In contrast, children with Down Syndrome exhibited higher supine AHI and RDI compared to those with CP (all *p* < 0.001). Awake arterial O_2_ saturation, average arterial O_2_ desaturation, Nadir of O_2_ saturation, and hypopnea were all significantly lower in patients with CP than in those with Down Syndrome (all *p* < 0.001).

Patients with neurological conditions were significantly more likely to have higher AHI categories (OR: 1.80; 95%CI: 1.12–2.91). When stratified by neurological condition, non-CP patients were significantly more likely to have higher AHI categories (OR: 2.57; 95%CI: 1.44–4.59). However, patients with CP had similar risk of AHI as controls (OR: 1.06; 95%CI: 0.57–1.98).

### 3.3. Quality-of-Life Parameters

**Figure 1:**
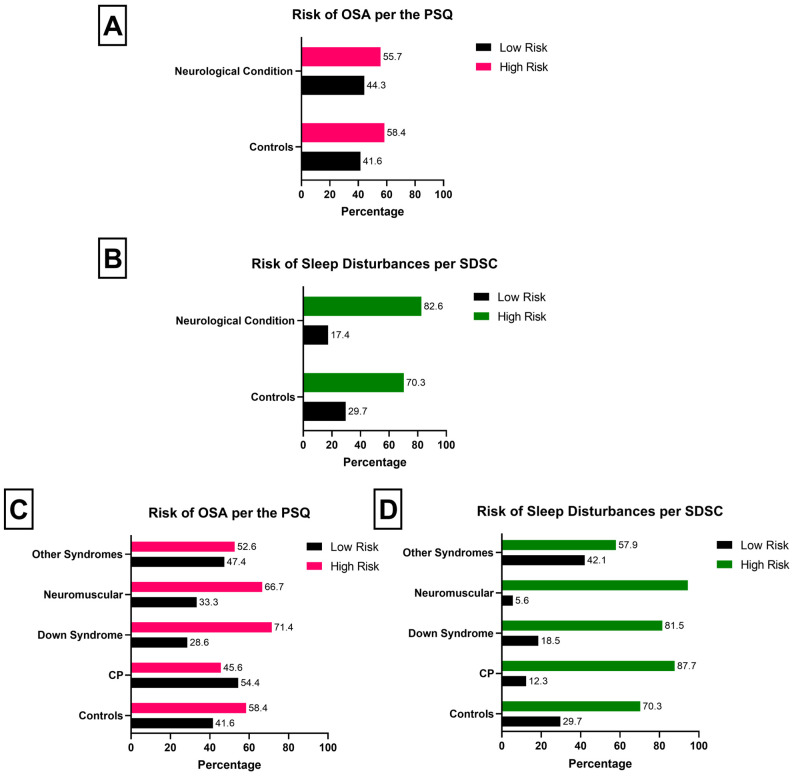
Demonstration of sleep disorder risk using the PSQ (**A**,**B**) and the SDSC questionnaire (**C**,**D**). Low risk per the PSQ (**A**,**C**) indicates a lower likelihood of sleep-disordered breathing [total score < 0.33]. High risk per the PSQ (**A**,**C**) indicates a higher risk of sleep-disordered breathing [total score > 0.33]. Low risk per the SDSC (**B**,**D**) indicates a lower likelihood of acute sleep disturbances [total score < 39], whereby high risk (**B**,**D**) indicates the opposite [total score > 39].

Risk of OSA according to the PSQ was insignificant for controls and patients with neurological conditions (OR: 0.898; 95%CI: 0.562–1.433; *p* = 0.720) (Figure 1A). Stratification by neurological condition did not yield significant differences (Figure 1B). Patients with neurological conditions had a significantly higher risk of sleep disturbances according to the SDSC questionnaire compared to controls (OR: 2.015; 95%CI: 1.015–3.998; *p* = 0.043) (Figure 1C). When stratified by neurological condition, only patients with CP demonstrated worse risk of sleep disturbances compared to non-neurological controls (OR: 3.022; 95%CI: 1.186–7.697) (Figure 1D). Patients with non-CP neurological conditions had a similar risk of sleep disturbances compared to controls.

## 4. Discussion

In this study, we found that patients with CP were more likely to experience sleep disturbances than controls. Among polysomnography parameters, only sleep efficacy and total sleep time differed significantly between patients with CP and non-neurological controls. When compared with children with Down Syndrome, however, patients with CP demonstrated a lower arousal index and AHI, but had significantly higher arterial O_2_ saturation, arterial O_2_ desaturation, and Nadir of O_2_ saturation.

Our findings are consistent with the published literature highlighting a higher likelihood of sleep disorders among patients diagnosed with neurological disorders, particularly epilepsy or CP [1,8]. In fact, studies speculate that patients with co-existing CP and epilepsy are at a higher risk of sleep disordered breathing [8,20]. The various factors contributing to sleep disorders among patients with CP include abnormal muscle tone, craniofacial abnormalities, intellectual disability, visual impairment, seizures, anti-epileptic medications, OSA, restricted movements, and chronic pain [21,22]. Children with more severe CP, as measured by the GMFCS, are at a higher risk of sleep disorders in general, and OSA more specifically [23]. This is attributed to the abnormal upper airway muscle tone, severe laryngomalacia due to reduced tone in the supraglottic structures, and concurrent pseudobulbar palsy associated with CP, which can impact sleep [24,25,26]. Dias et al. demonstrate that GMFCS severity class V might be associated with the highest risk of respiratory health issues in the form of high OSA prevalence [27]. Nonetheless, these findings were based on subjective sleep assessment tools that have not been validated among patients with CP.

It should be noted that the literature demonstrates significant variability in terms of sleep disorder risk and CP type or GMFCS severity class, as there are multiple reports denouncing type or severity-based differences [28,29,30]. Unfortunately, the aforementioned studies do not shed light upon this finding. We postulate that such differences are either due to sampling errors in which only a single-severity form of CP dominated the sample, or due to reduced statistical power considering the need to represent five distinct GMFCS groups. Furthermore, Romeo et al. hint that SDSC items pertaining to non-restorative sleep might not be relevant to children with severe motor disorders [31], indicating a potential limitation of tools and their capacity for sleep assessment in patients with CP.

However, our PSG findings were significantly more abnormal for non-CP patients compared to their CP counterparts. Patients with epilepsy, Down Syndrome, Duchenne Muscular Dystrophy, or Prader–Willi syndrome were significantly more likely to have worse clinical markers of sleep compared to patients with CP. Unfortunately, there is limited evidence on the magnitude of the effect of different neurological disorders on the quality of sleep among affected patients. However, it appears that more complicated neurological conditions are associated with worse sleep. For example, OSA and epilepsy have a bi-directional relationship [32]. OSA has been implicated in the worsening of epilepsy through chronic sleep deprivation and hypoxia. Conversely, electrical discharges impairing upper airway control have been implicated as a source of central apneas. In fact, it is postulated that sudden unexpected death in epilepsy is related to the relationship between seizures and airway control [32,33,34]. Duncan and Maitre argue that it is yet to be determined whether sleep disturbances among patients with CP are a result of the epileptogenic processes or the initial brain insult [13]. In the case of Duchenne Muscular Dystrophy, significant proximal weakness, chronic thoracic wall weakness and scoliosis, and long-term corticosteroid usage contribute to a compounding effect on quality of sleep [35].

It is also plausible to hypothesize that progressive neurological disorders are more likely to be associated with worse sleep compared to non-progressive disorders such as CP. However, isolating the direct link between neurological diseases and sleep quality is extremely challenging. The link between neurological and sleep impairment is multifaceted [13]. Such a link is influenced by both pathophysiological (intrinsic) mechanisms and environmental (extrinsic) factors [36,37,38,39]. Intrinsic factors such as abnormal EEG patterns, hypothalamic–pituitary axis impairment, and musculoskeletal limitations could all, theoretically, result in abnormal sleep [36,38,39,40]. Many of the aforementioned factors lead to the high severity of OSA in patients with Down Syndrome [41]. Nonetheless, a lack of optimization of extrinsic factors is crucial for both the affected patient and their associated caregiver [13].

Interestingly, the findings of literature exploring the association between CP and sleep quality should be interpreted with caution [42]. The vast majority of the literature uses either the SDSC or the PSQ to measure the association between CP and risk of sleep disturbance or OSA, respectively [43]. None of these tools were psychometrically validated in a cohort of children with CP. This is further complicated by the lack of psychometric validations for the aforementioned questionnaire across a variety of languages, including Arabic and English [44,45]. Similarly, these tools, in terms of diagnostic utility, are not externally validated in CP cohorts. According to Bautista et al., none of the available tools are applicable to children with CP, which limits their clinical applicability [43].

Our results should be interpreted within the context of a number of limitations. Firstly, the cross-sectional design limits our ability to identify any causal relationships. Similarly, the consecutive sampling approach may have introduced selection bias through over/under-representing certain clinical characteristics of included participants. Moreover, this form of sampling could have limited the generalizability of findings through the production of a clinically skewed sample (e.g., patients with severe motor impairment). Considering that all patients and their respective controls are recruited from a hospital-based setting could have skewed the results of the sleep study. This is relevant, as most of the controls were patients with asthma—a condition that is strongly correlated with sleep disorders. Secondly, due to resource limitations, the number of CP patients included within the study was disproportionately lower than the number of both control and non-CP neurological patients. This limitation could have impacted the statistical power of testing and influenced the direction of examined associations and mean differences. Also, it may have led to an imbalance of patients within the neurological disease cohort, as PSG testing was dependent on clinical referrals and was not voluntary. Thirdly, the study did not account for all factors influencing sleep quality, especially extrinsic factors. Finally, the study used the PSQ and SDSC questionnaires due to the lack of any other validated tools for the examination of sleep among patients with CP.

## 5. Conclusions

The above findings indicate that patients with neurological diseases are at a higher risk of sleep disturbances compared to controls. This was significantly more apparent in non-CP patients than in those with CP. At present, PSG remains the most objective and reliable tool for evaluating these disturbances, as the quality and psychometric properties of available sleep survey tools are limited in pediatric patients with specific neurological disorders, including CP.

## Figures and Tables

**Table 1:** Characteristics of included participants.

Variables	Controls	Neurological Patients
Age	8.5 (6.3, 11.5)	7.8 (4.3, 11.5)
Gender		
Male	115 (66.5%)	77 (62.6%)
Female	58 (33.5%)	46 (37.4%)
BMI		
Normal	66 (40%)	43 (39%)
Obesity	42 (25%)	22 (20%)
Overweight	42 (25%)	21 (19%)
Underweight	15 (9.1%)	23 (21%)
Unknown	8	14
Family History (present)	30 (17%)	8 (6.5%)
Consanguinity (present)	2 (1.2%)	7 (5.7%)

**Table 2:** Polysomnography findings for patients with neurological conditions and controls.

Parameters	Controls	Neurological Patients	*p*-Value
Sleep Efficacy	84 [73.0–89.0]	77 [69.0–86.0]	<0.001
Total Sleep Time	347.5 [310.5–381.5]	305 [256.0–352.0]	<0.001
Sleep Onset	26.5 [13.0–42.0]	19 [10.0–45.0]	0.376
N1	7 [4.0–10.0]	8 [5.0–16.0]	0.005
N2	48 [42.0–53.0]	47 [40.0–55.0]	0.972
N3	34 [28.0–41.0]	32 [25.0–39.0]	0.053
REM	10 [3.0–15.0]	6 [0.0–14.0]	0.072
Arousal Index	15 [9.0–22.0]	17 [11.0–27.0]	0.005
AHI	6 [3.0–14.0]	9.5 [4.0–19.0]	0.007
Central Apnea	6 [2.0–13.0]	5 [2.0–12.5]	0.616
Obstructive Apnea	1 [0.0–5.0]	1 [0.0–5.0]	0.409
Mixed Apnea	0 [0.0–2.0]	1 [0.0–2.0]	0.644
Hypopnea	19 [8.0–47.0]	37 [15.5–67.0]	0.001
Awake arterial O_2_ saturation	96 [95.0–97.0]	95 [94.0–96.0]	0.003
Average arterial O_2_ desaturation	95 [94.0–96.0]	94 [93.0–95.0]	0.085
Nadir of O_2_ saturation	87 [81.0–90.0]	84 [79.0–89.0]	0.037
Snoring time (mins)	13.5 [1.0–77.5]	17 [2.0–65.0]	0.763
Percentage of snoring	4 [0.3–22.0]	6 [0.5–25.0]	0.363
Duration (Supine)	243.1 [136.1–348.5]	245.95 [126.3–325.5]	0.608
REM (Supine)	8.8 [0.0–15.2]	3.4 [0.0–13.9]	0.207
NREM (Supine)	80 [72.1–88.7]	78.2 [65.1–86.3]	0.180
AHI (Supine)	5.5 [3.1–12.8]	9.2 [3.6–21.6]	0.093
RDI (Supine)	5.5 [3.1–12.8]	9.2 [3.7–21.6]	0.086
Duration (Right)	35.9 [6.3–98.4]	55.7 [8.7–94.3]	0.889
REM (Right)	0 [0.0–6.3]	0 [0.0–5.9]	0.708
NREM (Right)	80.55 [60.0–89.1]	70.8 [55.5–88.1]	0.278
AHI (Right)	2.55 [0.0–11.2]	10.1 [0.0–19.8]	0.070
RDI (Right)	2.55 [0.0–11.2]	10.1 [0.0–19.8]	0.070
Duration (Left)	78 [18.8–188.1]	82 [25.0–138.2]	0.822
REM (Left)	0 [0.0–13.6]	0 [0.0–0.2]	NA
NREM (Left)	76.2 [55.9–90.7]	83.9 [64.1–95.3]	0.196
AHI (Left)	3.9 [1.4–13.1]	6.6 [1.8–21.4]	0.413
RDI (Left)	3.9 [1.4–13.1]	6.6 [1.8–21.4]	0.413

**Table 3:** Polysomnography findings stratified by type of neurological condition.

Parameters	Controls	CP	Down Syndrome	Neuromuscular	Syndromes	*p*-Value
Sleep Efficacy	84 [73.0–89.0] *	78 [70.0–88.0] *	75 [67.0–82.0]	68 [55.0–80.0]	83 [76.0–89.0]	<0.001
Total Sleep Time	347.5 [310.5–381.5] *	317 [259.0–365.0] *!	294 [229.0–311.0] !	301 [225.0–362.0]	310 [266.0–372.0]	<0.001
Sleep Onset	26.5 [13.0–42.0]	16 [9.0–41.0]	20 [12.0–54.0]	35.5 [15.0–62.0]	15 [8.0–29.0]	0.278
N1	7 [4.0–10.0]	7 [4.0–16.0] *	8 [5.0–15.0]	13.5 [7.0–16.0] *	7 [6.0–16.0]	0.006
N2	48 [42.0–53.0]	46 [37.0–55.0]	52.5 [40.5–56.5]	46.5 [43.0–50.0]	51 [41.0–55.0]	0.544
N3	34 [28.0–41.0]	34 [24.0–41.0]	31 [25.5–37.5]	30.5 [26.0–37.0]	33 [22.0–36.0]	0.260
REM	10 [3.0–15.0]	5 [0.0–15.0]	6 [0.0–14.0]	9 [0.0–14.0]	7 [0.0–18.0]	0.440
Arousal Index	15 [9.0–22.0]	15 [9.0–22.0] *!	20 [15.0–33.0] *	16 [12.0–27.0]	21 [17.0–34.0] !	0.002
AHI	6 [3.0–14.0]	6 [3.0–14.0] *!	17 [11.5–39.0] *	8 [4.0–13.0]	11 [8.0–22.0] !	<0.001
Central Apnea	6 [2.0–13.0]	5 [3.0–10.0]	7.5 [2.0–18.0]	4 [2.0–12.0]	4 [1.0–17.0]	0.913
Obstructive Apnea	1 [0.0–5.0]	2 [0.0–5.0]	2.5 [0.5–13.5]	1 [0.0–2.0]	0 [0.0–2.0]	0.130
Mixed Apnea	0 [0.0–2.0]	1 [0.0–1.0]	1.5 [0.0–3.0]	0 [0.0–2.0]	0 [0.0–1.0]	0.750
Hypopnea	19 [8.0–47.0]	24 [8.0–54.0] *!	64.5 [36.0–115.0] *	33 [6.0–45.0]	55 [18.0–118.0] !	<0.001
Awake arterial O_2_ saturation	96 [95.0–97.0]	96 [94.0–96.0] *	94 [93.0–95.0] *	96 [95.0–96.0]	95 [94.0–96.0]	<0.001
Average arterial O_2_ desaturation	95 [94.0–96.0]	95 [93.0–96.0] *	93.5 [92.0–94.0] *	95 [93.0–96.0]	94 [93.0–95.0]	<0.001
Nadir of O_2_ saturation	87 [81.0–90.0]	86 [80.0–90.0] *	80 [72.0–86.0] *	85.5 [84.0–89.0]	82 [76.0–89.0]	0.004
Snoring time (mins)	13.5 [1.0–77.5]	10 [1.5–22.0]	32 [7.0–113.5]	31.5 [1.0–70.0]	44 [6.0–68.0]	0.183
Percentage of snoring	4 [0.3–22.0]	3 [0.5–9.0]	11.5 [2.5–36.5]	8 [0.2–29.0]	14 [2.0–27.0]	0.09
Duration (Supine)	243.1 [136.1–348.5]	205.8 [74.5–320.5]	239.1 [196.5–306.2]	333.5 [288.5–365.4]	198.35 [91.6–265.7]	0.078
REM (Supine)	8.8 [0.0–15.2]	1.45 [0.0–13.6]	5.6 [0.0–14.3]	0 [0.0–8.7]	6.35 [0.0–16.3]	0.517
NREM (Supine)	80 [72.1–88.7]	77.65 [58.8–86.3]	77.9 [69.7–83.6]	76.6 [65.1–82.7]	83.7 [70.6–92.4]	0.349
AHI (Supine)	5.5 [3.1–12.8]	4.4 [0.3–12.1] *	20.55 [15.5–40.4] *	6.3 [4.3–14.9]	9.05 [7.2–28.9]	<0.001
RDI (Supine)	5.5 [3.1–12.8]	4.9 [0.7–12.1] *	20.55 [15.5–40.4] *	6.3 [4.3–14.9]	9.05 [7.2–28.9]	<0.001
Duration (Right)	35.9 [6.3–98.4]	49.7 [7.5–84.1]	56.4 [6.9–91.0]	39.65 [9.9–69.9]	84.95 [10.5–95.8]	0.932
REM (Right)	0 [0.0–6.3]	0 [0.0–0.0]	0 [0.0–0.0]	0 [0.0–33.1]	0 [0.0–18.6]	0.682
NREM (Right)	80.55 [60.0–89.1]	63.4 [33.0–91.5]	76.2 [55.7–84.1]	59.2 [10.1–81.7]	74.05 [59.6–87.6]	0.679
AHI (Right)	2.55 [0.0–11.2]	2.3 [0.0–16.3]	11.9 [4.7–19.8]	1.9 [0.0–17.0]	16.45 [3.5–28.6]	0.117
RDI (Right)	2.55 [0.0–11.2]	2.3 [0.0–16.3]	11.9 [4.7–19.8]	1.9 [0.0–17.0]	16.45 [3.5–28.6]	0.117
Duration (Left)	78 [18.8–188.1]	89 [40.2–226.5]	56.4 [15.0–108.9]	38.3 [1.5–82.0]	82.3 [16.7–112.4]	0.426
REM (Left)	0 [0.0–13.6]	0 [0.0–6.1]	0 [0.0–0.0]	0 [0.0–0.0]	0 [0.0–10.4]	NA
NREM (Left)	76.2 [55.9–90.7]	84.8 [72.2–95.3]	71.2 [57.1–88.0]	99 [60.0–100.0]	84.1 [74.2–94.6]	0.282
AHI (Left)	3.9 [1.4–13.1]	6.5 [2.3–17.0]	9.75 [0.0–22.1]	4.2 [0.0–8.1]	7.8 [2.2–38.4]	0.791
RDI (Left)	3.9 [1.4–13.1]	6.5 [2.3–17.0]	9.75 [0.0–22.1]	4.2 [0.0–8.1]	7.8 [2.2–38.4]	0.791

* This annotation indicates significant differences between subgroups on post-hoc testing. ! This annotation indicates significant differences between subgroups on post-hoc testing.

## Data Availability

The datasets used and/or analyzed during the current study are available from the corresponding author.
